# A Novel Integrated Way for Deciphering the Glycan Code for the FimH Lectin

**DOI:** 10.3390/molecules23112794

**Published:** 2018-10-28

**Authors:** Tetiana Dumych, Clarisse Bridot, Sébastien G. Gouin, Marc F. Lensink, Solomiya Paryzhak, Sabine Szunerits, Ralf Blossey, Rostyslav Bilyy, Julie Bouckaert, Eva-Maria Krammer

**Affiliations:** 1 Danylo Halytsky Lviv National Medical University, Department of Histology, Cytology and Embryology & Department of Medical Biology, Parasitology and Genetics, 79010 Lviv, Ukraine; tetiana.dumych@gmail.com (T.D.); sola.paryzhak@gmail.com (S.P.); r.bilyy@gmail.com (R.B.); 2 University of Lille, CNRS UMR8576 UGSF, Institute for Structural and Functional Glycobiology, F-59000 Lille, France; clarisse.bridot@univ-lille.fr (C.B.); marc.lensink@univ-lille.fr (M.F.L.); ralf.blossey@univ-lille.fr (R.B.); 3 Chimie Et Interdisciplinarité, Synthèse, Analyse, Modélisation, UMR 6230 Centre National de la Recherche Scientifique, Université Nantes, 44322 Nantes, France; Sebastien.Gouin@univ-nantes.fr; 4 Univ. Lille, CNRS, Centrale Lille, ISEN, Univ. Valenciennes, UMR 8520-IEMN, F-59000 Lille, France; sabine.szunerits@univ-lille.fr

**Keywords:** Manα1,3Man, Manα1,2Man, FimH, binding mode, Enzyme-Linked LectinoSorbent assay, microcalorimetry, molecular dynamics, thermodynamics, entropy, high-mannose *N*-glycan

## Abstract

The fimbrial lectin FimH from uro- and enteropathogenic *Escherichia coli* binds with nanomolar affinity to oligomannose glycans exposing Manα1,3Man dimannosides at their non-reducing end, but only with micromolar affinities to Manα1,2Man dimannosides. These two dimannoses play a significantly distinct role in infection by *E. coli*. Manα1,2Man has been described early on as shielding the (Manα1,3Man) glycan that is more relevant to strong bacterial adhesion and invasion. We quantified the binding of the two dimannoses (Manα1,2Man and Manα1,3Man to FimH using ELLSA and isothermal microcalorimetry and calculated probabilities of binding modes using molecular dynamics simulations. Our experimentally and computationally determined binding energies confirm a higher affinity of FimH towards the dimannose Manα1,3Man. Manα1,2Man displays a much lower binding enthalpy combined with a high entropic gain. Most remarkably, our molecular dynamics simulations indicate that Manα1,2Man cannot easily take its major conformer from water into the FimH binding site and that FimH is interacting with two very different conformers of Manα1,2Man that occupy 42% and 28% respectively of conformational space. The finding that Manα1,2Man binding to FimH is unstable agrees with the earlier suggestion that *E. coli* may use the Manα1,2Man epitope for transient tethering along cell surfaces in order to enhance dispersion of the infection.

## 1. Introduction

Glycans on proteins and lipids in the plasma membrane play crucial roles in cell-cell and cell-pathogen recognition and binding. Adhesion of pathogenic *Escherichia coli* (*E. coli*) to host cells is the result of the interaction of type 1 fimbriae with high-mannosylated glycoprotein (MGP) receptor molecules exposed on the surface of epithelial cells located in the oropharyngeal, gastrointestinal and urinary tract [1,2,3]. At the molecular level this interaction is achieved by the bacterial lectin FimH, located at the top of type 1 fimbriae, which specifically binds the terminal α-d-mannose sugars exposed by the MGPs. The same sugar (α-d-mannose) can be used to inhibit type-1 fimbriae-dependent bacterial adhesion [4]. Synthetic mannose derivatives, such as the heptyl α-d-mannopyranoside, have shown to inhibit FimH adhesin even more effectively [5,6,7,8,9]. The need for such compounds is high as FimH-mediated binding of *E. coli* is of central importance in a variety of diseases including Crohn’s Disease (CD), urinary tract infections (UTI), enteritis, diarrhoea, sepsis and meningitis [10].

FimH from uropathogenic *E. coli* strains (UPEC), adherent and invasive *E. coli* strains (AIEC), evidenced to be involved in the development of CD [11,12,13], as well as from other *E. coli* strains, consists of two immunoglobulin (Ig)-like domains—the lectin or carbohydrate recognition domain (amino acids (aa.) 1–157), communicating via a short flexible linker made by Thr158, Gly159 and Gly160 with the pilin domain (aa. 161 to 276) that connects FimH to the other pilins forming the fimbrial rod [14,15]. FimH was reported to adhere to the MGP carcinoembryonic antigen-related cell adhesion molecule 6 (CEACAM6) overexpressed and exposed on epithelial cells of the gastrointestinal tract in the case of CD and AIEC [13,16] and to the MGP Uroplakin Ia (UPIa), present on the surface of epithelial umbrella cells of the urinary tract, in the case of UTI and UPEC [17]. UPIa was shown to contain exclusively high-mannosylated *N*-glycans with 6, 7, 8 and 9 terminally exposed mannose residues [18]. Human CEACAM6 was shown to contain at least two high-mannosylated *N*-glycosylation sites with 5, 6, 7, 8 and 9 terminally exposed mannose residues [19,20,21].

Both UPIa and CEACAM6 glycans carry Manα1,3Man-terminating *N*-glycans, that can be shielded by additional non-reducing Manα1,2Man endings depending on the oligomannoside identity. The two epitopes (Manα1,2Man and Manα1,3Man) have thus been suggested to have different functions in infection by *E. coli*: Manα1,2Man might be used for the bacteria to spread during the infection and by the host to shield the Manα1,3Man epitope in its glycosylated proteins. The Manα1,3Man epitope might be more relevant to strong bacterial adhesion and invasion [22]. The FimH lectin was also reported to bind to isolated high-mannosylated *N*-glycans with micro- to nanomolar affinity depending on whether these glycans exposed at their non-reducing end an α-1-2 (Manα1,2Man) or α-1-3 (Manα1,3Man) linked dimannoside, with a preference for the latter [23,24,25]. Surface Plasmon Resonance (SPR) measurements indicated that the preference for Manα1,3Man is kept even when the isolated dimannoses are studied and that this preference depends on the clinical *E. coli* strain and/or of the FimH variant [24]. However, the difference in affinity of the two isolated dimannoses is less pronounced compared to the same non-reducing epitopes of the oligomannoses [25].

In this manuscript we combine the Enzyme-Linked LectinoSorbent Assay (ELLSA) and Isothermal Titration Calorimetry (ITC) measurements of the FimH-dimannose interactions with different molecular simulation tools to understand at a molecular level the difference in binding affinity for Manα1,2Man and Manα1,3Man. The integration of these analytical methods allows for a novel way of deciphering the glycan code of FimH.

## 2. Results and Discussion

### 2.1. Experimentally Determined Binding Affinities Highlight a Higher Affinity of Fimh Towards Manα1,3Man

**Figure 1:**
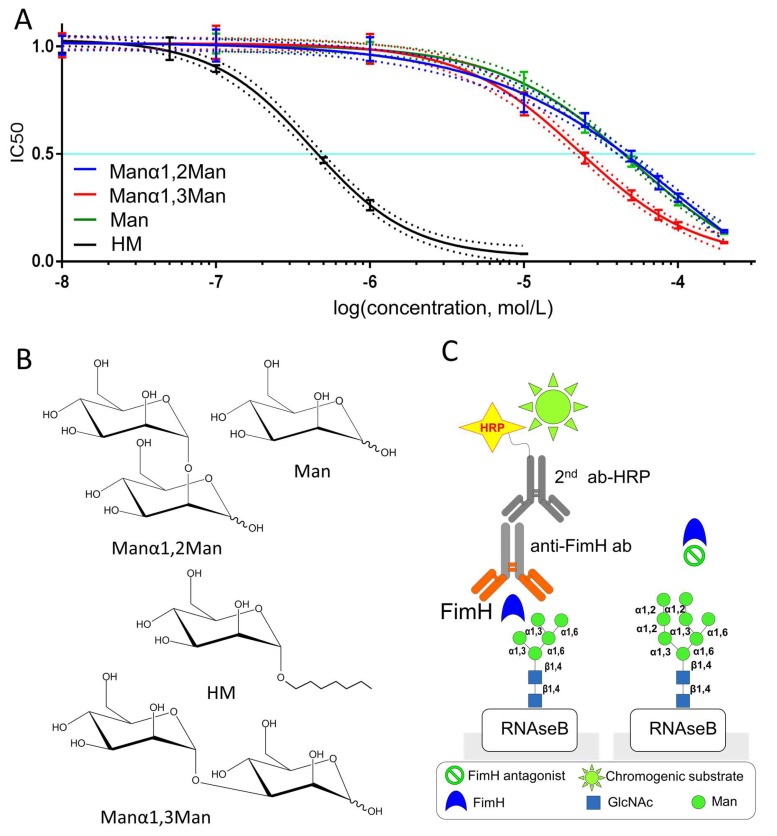
IC_50_ measurements of different mannosides towards their ability to block FimH interaction with oligomannose glycoepitopes using ELLSA assay. (**A**) Interaction of FimH with oligomannose glycoepitopes depends on mannoside concentration. The chemical structures of the tested mannosides are shown in (**B**,**C**) In ELLSA, the high-mannose *N*-glycan on RNAseB (shown are oligomannose-5 (left) and oligomannose-8 (right)) is used as target for FimH lectin binding [26,27]. The latter is detected by incubation with anti-FimH antibodies (Anti-FimH ab) and secondary horseradish peroxidase HRP-conjugated antibodies (2nd ab-HRP). Horseradish peroxidase activity is visualized by chromogenic substrate (TMB, 3,3’,5,5’-tetramethylbenzidine). The concentration of inhibitor needed to inhibit 50% of FimH binding to RNAseB corresponds to the IC_50_.

Bovine ribonuclease B, or RNAseB, is a good binder of the FimH adhesin because it carries a single high-mannosylated *N*-glycan of which the major glycoform is oligomannose-5, carrying two Manα1,3Man endings (Figure 1C) [26,27]. RNAseB was thus used in the ELLSA approach to measure the IC_50_ for different mannosides displayed in Figure 1B that compete with RNaseB for FimH binding. The so determined IC_50_ showed that the dimannose Manα1,2Man binds with about the same affinity as the α-d-mannose (Man; see Table 1), indicating that an additionally α-1,2 linked mannose does not prove beneficial for binding. In contrast, the addition of α-1,3 linked mannose (leading to Manα1,3Man), leads to an about 2.5-fold increase of affinity. This is in line with previous SPR experiments using FimH proteins from different *E. coli* strains, which showed an increased affinity of FimH towards Manα1,3Man [24]. It is remarkable that no complete inhibition of binding between FimH and the low-affinity sugars α-d-mannose and Manα1,2Man could be achieved, in contrast to for Manα1,3Man and HM (Figure 1A). ITC experiments done in parallel show comparable results, an about 3-fold reduced affinity for Manα1,2Man compared to Manα1,3Man (Table 1). Independent of the used techniques, the Manα1,3Man binding is more than 10 times weaker compared to the well-studied FimH-inhibitor HM [28,29,30,31].

**Figure 2:**
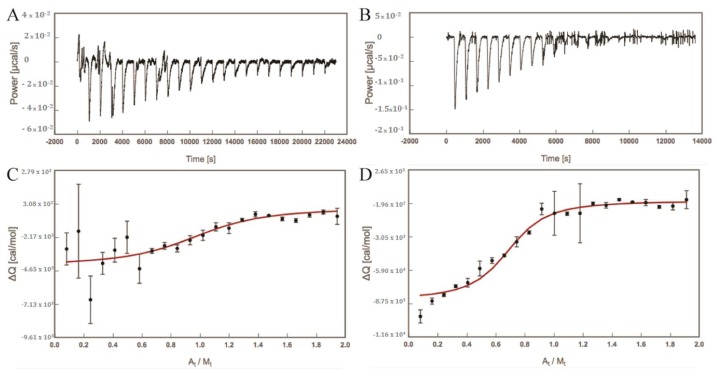
Enthalpogram of the dimannose (**A**) binding to FimH [M]. Processed data (**A**,**B**) and their integrated signal (**C**,**D**) for Manα1,2Man (**A**,**C**) and Manα1,3Man (**B**,**D**). ΔQ = ΔH at constant pressure, A_t_/M_t =_ ligand/protein molar ratio. For more detail, see Section 3.2.

We further used ITC measurements to determine the thermodynamic behaviour of the differential dimannose binding to FimH (see Figure 2). Manα1,3Man displays clearly an almost totally enthalpy-driven binding (see Table 1), in agreement with previously reported results for other mono- and oligomannoside derivatives [32,33] and for a series of biphenyl α-d-mannosides [34,35]. In contrast, the enthalpic contribution to the binding of Manα1,2Man is much lower compared to the other mannosides and with a larger the uncertainty (see Table 1). 

Moreover, the gain in entropy is much larger for Manα1,2Man compared to the other compounds and is getting close to the enthalpic contribution. Remarkably, despite the smaller heat signal (ΔQ) of the exothermal reaction between Manα1,2Man and FimH, a significantly longer time for Manα1,2Man than for Manα1,3Man was needed to return to an equilibrium, where equilibrium is indicated by a return the baseline where no further heat was produced (zero power (µcal/s); see Figure 2A,B). Therefore, the time spacing between injections of Manα1,2Man into the measurement cell containing FimH was doubled compared to for Manα1,3Man.

### 2.2. Computed Binding Affinities Concur with Experimental Data

The binding affinities of the dimannoses towards the FimH lectin were also determined using an *in-silico* approach: initial binding poses for Manα1,2Man and Manα1,3Man were obtained by molecular docking (see Section 3.3) and subjected to molecular dynamic (MD) simulations (see Section 3.4). The free binding energies ΔG_binding_ were determined using a Molecular Mechanics Poisson-Boltzmann Surface Area (MM-PBSA) single trajectory approach (see Section 3.5). For comparison the binding affinities of HM and Man were also computed (see Section 3.3 and Section 3.5). In agreement with the ITC measurements (see Table 1) the ΔG_binding_ (see Table 2) energies show the same trend: HM has the highest binding affinity for FimH, followed by Manα1,3Man and Manα1,2Man, and Man has the lowest affinity for FimH. The decomposition of the free energy ΔG_binding_ clearly highlight that three effects distinguish the binding of the different compounds. One is the electrostatic energy contribution ΔE_ele_, which is significantly higher for Manα1,3Man compared to the other tested ligands. The second contribution is the van-der-Waals contribution ΔE_vdw_, which is much lower for Man compared to the other three compounds. The difference in the ΔE_vdw_ contribution most likely originates from the size of the ligand. Man is much smaller compared to the other ligands and is thus not able to form van-der-Waals interactions with the hydrophobic rim of the binding pocket. Favourable interaction of FimH inhibitors with the tyrosine gate have been shown to significantly contribute to their binding affinity [9]. The third contribution is the polar solvation energy contributions (ΔG_solv POLAR_), which is much higher for Manα1,3Man compared to the other molecules, indicating a higher preference for Manα1,3Man to remain in solution.

### 2.3. Molecular Details of Dimannose Binding to FimH

**Figure 3:**
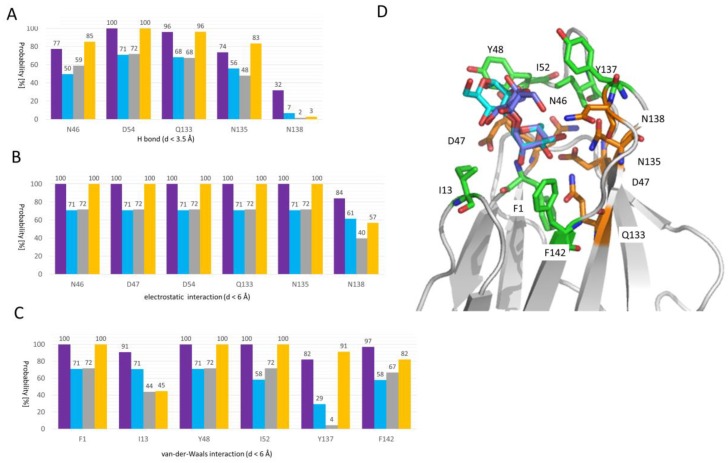
FimH-dimannose interactions as extracted from the MD simulations. The probability of finding a FimH binding site residue within (**A**) H bond, (**B**) electrostatic interaction, and (**C**) van-der-Waals interaction distance (**D**) of the Manα1,2Man (lilac), Manα1,3Man (cyan), Man (grey), and HM (orange) ligand is plotted against the residue number. Only residues with a probability of at least 25% for at least one ligand are shown. For more details regarding the calculations see Section 3.4. (**D**) The position of the different listed residues in (**A**–**C**) is highlighted in the FimH binding site residue within H bond and/or electrostatics interaction range are depicted as red sticks onto the Manα1,2Man (lilac) and Manα1,3Man (cyan) FimH conformation representative for cluster #1 (see Figure 4 and Figure 5) and residues within van-der-Waals interaction range are shown as orange sticks on the same structure (white cartoon).

The good qualitative agreement between experimentally measured and computationally determined affinities highlights the meaningfulness of the MD simulations (compare Table 1 and Table 2). We thus used them further to decipher the binding mode(s) of Manα1,2Man and Manα1,3Man at a molecular level. These two mannosides are of particular interest as they feature a different binding affinity but do only differ in the location of one single bond: in Manα1,2Man the two sugar molecules are α1-2 linked whereas they are α1-3 linked in the case of Manα1,3Man. The MD trajectories in which the sugars are bound in the FimH binding site were screened for possible hydrogen (H) bonds, electrostatic interactions and van-der-Waals interactions (see Figure 3 and Section 3.5). A first observation is that both Manα1,2Man and Manα1,3Man are close to the same set of residues. There are several polar residue side chains within H bond distance (namely Asn46, Asp54, Gln133, Asn135 and to a lesser extend Asn138; see Figure 3B), most of which are located at the bottom of the FimH mannose binding site and are coordinating the non-reducing mannose ring (see Figure 3D). These residues, if not engaged in a H bond, form an electrostatic interaction with the dimannoses (see Figure 3B). Asp47, placed at a longer distance from the non-reducing mannoside ring (see Figure 3D), can also engage in electrostatic interactions but not in a H bond (compare Figure 3B to Figure 3C). The here mentioned residues have been shown to form a polar binding pocket accommodating the mannose-ring of different FimH inhibitor and the natural epitopes [23,28,29,36]. Furthermore, most of these residues (Asn46, Asp47, Asp54, Gln133, Asn135, and Glu140) are invariant throughout all know strains of *E. coli* [37] and the mutation of any of these residues led to a loss of mannose binding and diminished virulence [38], further highlighting their importance in the binding process.

Both dimannoses, although to varying extent, can perform van-der-Waals interactions with Phe1, Ile13, Tyr48, Ile52, Tyr137 and Phe142 (see Figure 3C). These residues form collar of hydrophobic residues surrounding the FimH binding site [9,28,39,40]. The crystal structure of FimH with the branched oligomannose-3 [36] highlights the particular importance of the tyrosine gate formed by the residues Tyr48, Ile52 and Tyr137, for the binding of the mannose rings adjacent to the first mannose ring bound in the pocket (see Figure 3B). In line with this observation, also the here studied dimannoses interact by means of the reducing mannose ring within the tyrosine gate (see Figure 3C).

Interestingly both dimannoses also form van-der-Waals interaction with I13 even so with varying extent (about 100% for Manα1,2Man and about 50% for Manα1,3Man). This residue is located in the clamp loop, which undergoes a major conformational change when FimH forms high-affinity catch bonds with mannosides [41] and changes from its conformation to a high affinity state. Only a few examples exist so far, where a chemically engineered mannoside orients towards Ile13 [39,42], even so as a minor populated conformation. It is possible that natural ligands do form the interaction with Ile13 as a first step to trigger the conformational change.

Our data clearly indicate that both mannosides are within the vicinity of the same residues, however, Manα1,2Man with a higher persistence than Manα1,3Man. We additionally computed the same interaction profile for Man and HM (see Figure 3). In agreement with previous data [9], HM engages in van-der-Waals interactions with the tyrosine gate residues (Tyr48, Ile52, and Tyr137). Overall, the HM interaction profile is very similar to that observed of Manα1,2Man, which is in good agreement with the similar interaction energies for both compounds (ΔE_int_, see Table 2). As Man is a shorter molecule, it features less hydrophobic interactions in agreement with a less favourable van-der-Waals interaction energy in our free energy calculations (ΔE_vdw_, see Table 2). Even so Manα1,2Man features higher probabilities to be within the good range to perform electrostatic interactions with several protein residues (see Figure 3), these interactions must be less strong compared to the ones performed by Manα1,3Man as the electrostatic interaction energy (ΔE_ele_; see Table 2) favours strongly Manα1,3Man compared to Manα1,2Man. Also Manα1,3Man seems to behave more Man compared to Manα1,2Man, especially regarding the H bond and electrostatic interactions.

### 2.4. Manα1,3Man Finds a Stable Binding Position

We further analysed our Manα1,3Man MD trajectories in water and bound to FimH, to gain a deeper understanding of Manα1,3Man binding to FimH. We extracted from our MD trajectories the major conformation(s) of Manα1,3Man in the protein and in solution using a clustering algorithm, that identifies structurally similar ligand conformations along a single or several MD trajectories and determines the populations of these similar conformations (for more details see Section 3.5). 

**Figure 4:**
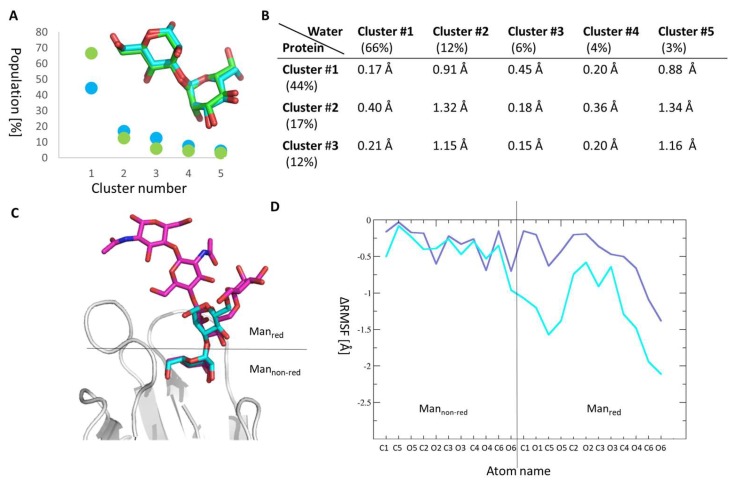
Molecular details of Manα1,3Man binding to FimH. (**A**) Clusters featuring similar structures were extracted from the MD simulations of Manα1,3Man in the FimH binding site (cyan) and in water (green) and plotted against their population occurrence (in %). Only the five highest ranking clusters are depicted, which account to 89% and 86% of the conformations in water and in the protein, respectively. As an inlay the superposition of the representative structure from cluster #1 in the protein (cyan) and in water (green) is shown. (**B**) RMSD values between the representative conformation of each cluster in water and in the protein. (**C**) Overlay of the presentative structure of Manα1,3Man (cyan) in cluster #1 of the Manα1,3Man·FimH complexes against the Oligomannose-3 (magenta) in its crystal structure (PDB ID: 2VCO). (**D**) The difference in root mean square fluctuations (ΔRMSF) is between the Manα1,2Man (lilac) and Manα1,3Man (cyan) in water and inside the FimH binding site is plotted against the atom name of the dimannose. The position of the first and second mannose ring in FimH is highlighted in the protein structure in (**C**).

In the protein as well as in water (see Figure 4A), there is one major cluster of structures, which accounts to 44% and 66% of all recorded conformations along the MD trajectories. As shown by the root mean square derivations (RMSD) between the representative structure of each cluster obtained in the protein and water (see Figure 4B), the clusters are all rather similar and the representative conformation in protein and in solution are very similar indeed (see Figure 4A inlay). All clusters feature an open, elongated Manα1,3Man as shown by the representative conformation of cluster #1 inside the protein binding site (see Figure 4C). The open Manα1,3Man conformation also nicely overlap with the oligomannose-3 FimH complex [36], which carries a Manα1,3Man at its non-reducing end (see Figure 4C). Furthermore, Manα1,3Man loses significantly in flexibility upon binding to FimH as evidenced by the relative root-mean-square-fluctuations (ΔRMSF; see Figure 4D). The loss in flexibility is not in agreement with the slight gain of entropy upon binding in the ITC measurements (see Table 1). This difference might indicate that other factors such as (de-)solvation effects and water orientations might important in the Manα1,3Man binding process. The loss in flexibility as well as a single major populated cluster of Manα1,3Man in the FimH binding site, indicates, that Manα1,3Man has a well-defined conformation in the FimH binding site, in which it forms several strong interactions with protein residues. This is in line with a favorable electrostatic binding energy in our MM-PBSA calculations (see Table 2) as well as the identified residues likely to interact with FimH (see Figure 3).

### 2.5. Molecular Reason for the High Entropic Gain of Manα1,2Man upon Fimh Binding

**Figure 5:**
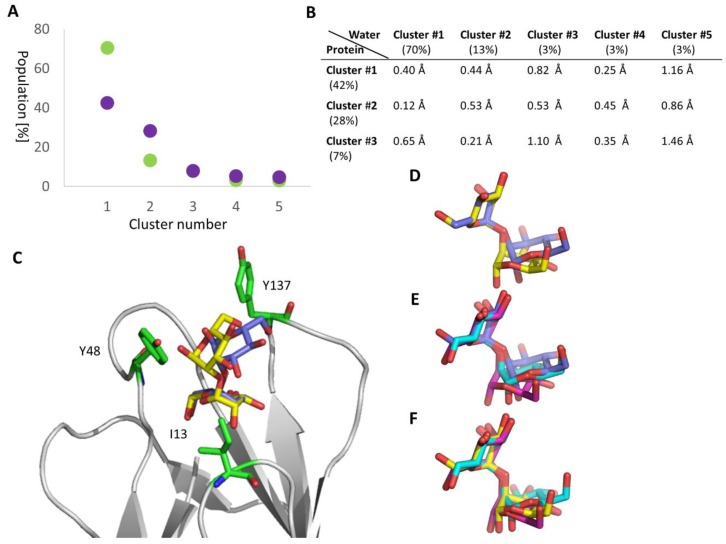
Molecular details of Manα1,2Man binding to FimH. (**A**) Clusters featuring similar structures were extracted from the MD simulations of Manα1,2Man in the FimH binding site (lilac) and in water (green) and plotted against their population occurrence (in %). Only the five highest ranking clusters are depicted, which account to 95% and 89% of the conformations in water and in the protein, respectively. (**B**) RMSD values between the representative conformation of each cluster in water and in the protein. (**C**) Overlay of the representative Manα1,2Man·FimH complex structure from cluster #1 (lilac) and #2 (yellow). A few residues representative of the FimH binding site are depicted additionally (green). For all residues interacting with Manα1,2Man see Figure 3. The protein is shown in white cartoon. To perform the overlay, the protein was superimposed. (**D**) Overlay of the cluster #1 (lilac) and #2 (yellow) of Manα1,2Man in the Manα1,2Man·FimH complex. To perform these and the following overlays, the non-reducing mannose was superimposed. (**E**) Overlay of cluster #1 (lilac) of the Manα1,2Man·FimH complex with the cluster #1 (magenta) and #4 (cyan) of Manα1,2Man alone in solution. (**F**) Overlay of cluster #2 (yellow) of the Manα1,2Man·FimH complex with the cluster #1 (magenta) and #4 (green) of Manα1,2Man alone in solution.

For Manα1,2Man, we observed two major clusters in the FimH binding site (cluster #1: 42% and cluster #2: 28%, see Figure 5A) in contrast to the situation in water, where there is only one highly populated cluster (cluster #1: 70%, see Figure 5A). Each of these clusters features a bracket-shaped Manα1,2Man conformation similar to what is observed in crystal structures of the dimannose Manα1,2Man or modified Manα1,2Man-dimannoside protein complexes, e.g., actinohivin (PDB ID: 4DEN) [43], PAL lectin (PDB ID: 1Q8O) [44], concanavaline A (PDB ID: 1BXH) [45], and langerin (PDB ID: 3P5F) [46]. In the bracket-shaped conformation the two mannoses face each other, which is in contrast to what is observed for Manα1,3Man, which features a more elongated, open conformation (see Figure 4C).

The two majorly populated clusters of Manα1,2Man bound to FimH differ significantly in their orientation in the binding site (see Figure 5C) and in their internal conformations (see Figure 5D), however, their psi and phi torsion angles fall within the low-energy region of the potential energy map for this disaccharide, as computed using the CARP webserver [47]. Furthermore, the conformation of Manα1,2Man in cluster #1 in solution does not represent the major cluster #1 in the protein as shown by the RMSD analysis (see Figure 5B). This cluster is rather similar to cluster #4 in the solution (see Figure 5E) and the major cluster #2 in the FimH binding site is closer to cluster #1 in solution (see Figure 5F). This indicates that Manα1,2Man does change its conformation when binding from solution to FimH and ones bound it seems to have difficulties to find the proper binding positions, wobbling between two major conformations. Manα1,2Man thus remains more flexible and settles less well in the FimH binding site compared to Manα1,3Man, which finds a tight binding position (see Figure 4). The higher flexibility of Manα1,2Man compared Manα1,3Man to can also be seen in the ΔRMSF data (see Figure 4B). Taken together, the remaining flexibility of Manα1,2Man in the FimH binding pocket and its alternative binding modes could explain the positive entropy contribution to the binding of FimH as measured using ITC. The finding that Manα1,2Man binding in the tyrosine gate of FimH is unstable and of a highly mobile nature agrees with the very long equilibration times in the microcalorimetry study and the lack of total inhibition in the ELLSA study. Previously we showed the central role of dispersion interactions in binding of FimH inhibitors [29]. Similarly, low-affinity inhibitors such as mannose have been shown to enhance bacterial traffic rather than concentrate them on the cells [36]. Our data are in line and agrees with the earlier suggestion [22] that *Escherichia coli* may use the Manα1,2Man epitope for transient tethering along cell surfaces in order to enhance dispersion of bacteria during infection.

## 3. Materials and Methods

### 3.1. Enzyme-Linked Lectinosorbent Assay

Immunosorbent microplates Nunc Maxisorp (Thermo Fisher Scientific, Waltham, MA, USA) were coated with 100 µL of 5 mg/mL solution of RNAse B in 100 mM carbonate/bicarbonate buffer, pH 9.6. Plates were incubated at 4 °C overnight and then washed (300 µL/well) three times with 1X phosphate-buffered saline (PBS) containing 0.05% Tween-20 (PBST). All wells were blocked with 200 µL 3% bovine serum albumin (BSA) in PBST and incubated at 37 °C 2 h. Then washed three times with PBST. Mannosides were dissolved in PBST to the appropriate concentrations, and added to microwells. FimH purified from *E. coli* [36] was diluted in PBST to 1 μg/mL and added to each well of plate and incubated for 1 h at room temperature. Wells were washed three times with PBST and incubated with 100 µL of rabbit anti-FimH antibodies IgG (aFimH) diluted 1:5000 in PBST for 1 h at room temperature. Then wells were washed three times with PBST and incubated with 100 µL of goat-anti-rabbit horseradish peroxidase HRP-labeled secondary antibody (Enzo Life Sciences Farmingdale, NY, USA) ( diluted 1:10,000 in PBST for 1 h at room temperature. Then washed three times with PBST and 100 µL of TMB (3,3′,5,5′-tetramethylbenzidine) containing H_2_O_2_ as a substrate were added to each well and incubated in darkness for 5–15 min. The reaction was stopped with 100 µL/well of 2 N sulfuric acid. Plate absorbance was analysed at 450 nm using a microplate BioAssay ReaderHTS7000 (Perkin Elmer, Waltham, MA, USA).

The IC_50_ value is given by the small molecule concentration, that is needed to inhibit FimH binding by 50 %. It was calculated for each compound using GraphPad Prism Software (GraphPad Software, La Jolla, CA, USA).

### 3.2. Isothermal Titration Calorimetry

The thermodynamic parameters of the interactions between FimH and the dimannoses Manα1,2Man, and Manα1,3Man were measured by isothermal titration calorimetry (ITC). The protein was dialyzed overnight at 4 °C against assay buffer using Slide-A-Lyzer dialysis cassettes with 10 kDa cut-off (Thermo Fisher Scientific, Waltham, MA, USA). All measurements were performed with a MicroCal^TM^ VP-ITC instrument (GE Healthcare, Northampton, MA, USA; sample cell volume of 1.4523 mL) at 25 °C, 307 rpm stirring speed, and 10 µcal/s reference power. For Manα1,3Man, 150 μM of ligand (A) was injected into 15.88 μM (M) of FimH lectin domain. For Manα1,2Man, 150 μM of ligand (A) was injected into 15.62 μM (M) of FimH lectin domain. Ligands were injected in 10 µL steps (22 injections in total) with a spacing of 5 min for Manα1,3Man and 10 min for Manα1,2Man, to ensure there were no overlapping peaks (Figure 2). Sigmoidal binding curves with complete saturation at the end of each experiment were obtained. Fitting was performed using AFFINImeter 1:1 interaction. Parameters ΔG (free energy change) and ΔS (entropy change) were calculated by introducing the measured ΔH and K_A_ into the formula:

ΔG = ΔH − TΔS = −RTlnK_A_ = RTlnK_D_ (1)

with T being the absolute temperature (295.15 K for the measurement and *R* the universal gas constant (8.314 J mol^−1^ K^−1^).

### 3.3. Induced Fit Docking

Docking experiments were performed using the GOLD software (The Cambridge Crystallographic Data Centre, Cambridge, UK). The six heavy atoms of the mannose ring of the ligand HM found in the coordinate file (PDB entry 4BUQ [29]) were used as a scaffold in the active site. A single internal structural water (below the O2 of the mannose ring of HM) in the active site was treated explicitly. The side chains of ten residues interacting with the mannose of HM in the binding site: Ile13, Asn46, Tyr48, Glu50, Asp54, Arg98, Gln133, Tyr137, Asn138, and Asp140, were allowed to adopt different conformations during the docking procedure. Starting conformations of the Manα1,2Man and the Manα1,3Man ligand were retrieved from the PDB database (Manα1,2Man: PDB ID 1Q8O [44]; Manα1,3Man: PDB ID: 1Q8P [44]). For each ligand, 10 docking poses that were energetically reasonable were kept while searching for the correct binding mode of the ligand. The decision to keep a trial pose was based on a computed energy for the interaction of the ligand with receptor of that pose. The ChemPLP fitness scoring function is the default in GOLD version 5.2 used to rank poses. Discovery Studio Visualizer 4.1 (Accelrys, San Diego, CA, USA) was used for viewing.

### 3.4. Molecular Dynamics Simulation

All molecular dynamics (MD) trajectories were generated in the isothermal-isobaric ensemble at 300 K with the program NAMD2.12 (Theoretical and Computational Biophysics Group in the Beckman Institute for Advanced Science and Technology, University of Illinois, Urbana-Champaign, USA) [48] using the CHARMM36 force field [49,50,51,52,53]. Long-range electrostatic interactions were calculated using the particle-mesh Ewald method [54]. A smoothing function was applied to truncate short-range electrostatic interactions. The Verlet-I/r-RESPA multiple time-step propagator [55] was used to integrate the equation of motions using a time step of 2 and 4 fs for short- and long-range forces, respectively. All bonds involving hydrogen atoms were constrained using the Rattle algorithm [56].

The best scoring pose of each docked dimannose·FimH complex (see Section 3.3) was used as initial coordinates for the MD simulations. FimH in complex with Man was generated by cutting the ligand Manα1,2Man after the first mannose. Each system (Manα1,2Man·FimH, Manα1,3Man·FimH, and Man·FimH) was solvated and the ionic concentration was set to 0.15 M NaCl. All ionizable groups were assigned their standard protonation state as predicted by propKa [57]. In total each molecular system comprised about 45,000 atoms. The equilibration was performed in three steps: (1) a 2.5 ns long equilibration of the solvent (water and ions) (2) 2.5 ns long equilibration in which only the protein backbone was fixed, and (3) an unrestrained 2.5 ns long simulation were performed. This was followed by 3 independent 50 ns long MD production trajectories for each system. Additionally, a simulation of the ligands Man, Manα1,2Man and Manα1,3Man alone in water were performed for 50 ns.

### 3.5. Trajectory Analysis

Hydrogen (H) bond, electrostatic and van-der-Waals interactions were determined using a distance criterion. A H bond was counted if a protein sidechain oxygen or nitrogen atom was within 3.5 Å of at least one of the oxygen atoms of the dimannosides. An electrostatic interaction was counted if a protein sidechain oxygen or nitrogen atom was within 6 Å of at least one of the oxygen atoms of the dimannosides. A van-der-Waals interaction was counted if at least a single protein carbon atom was within 6 Å of at least one of the carbon atoms of the dimannosides.

The flexibility of the Manα1,2Man and Manα1,3Man bound to FimH was determined using the root mean square fluctuation (RMSF) difference (ΔRMSF) of the ligands in the protein compared to its flexibility in water. The RMSF is a quantity describing the movement of each considered atom around the average structure and is defined as:

(2) $$\;{\mathrm{RMSF}}_{p}=\sqrt{\frac{1}{N}\overset{N}{\underset{n=1}{\sum }}{\left(p_{n}-\;\bar{p}\right)}^{2}}\;$$

where $p_{n}$ is the position of an atom of interest in the frame n, $\bar{p}$ is the position of the same atom in the average structure, and N is the total number of frames of a considered MD trajectory. As the moiety of the first mannose-ring of both ligands remains in a similar position during the trajectories, the RMSF was computed after alignment of the mannose ring using VMD [58].

The most abundant conformations of the dimannosides in water and within the protein were were determined by clustering the here produced MD trajectories using the G_CLUSTER tool of the MD suite GROMACS [59], based on the root mean square deviation (RMSD) matrix of the dimannose. Prior to the calculations the non-reducing end of the dimannose was aligned (water simulations) or the two tyrosine gate residues (Tyr48, and Tyr137) were aligned (dimannose·FimH simulations). A total of 5000/15,000 frames was used for the clustering of the water/FimH MD trajectories, extracted every 10 ps using the GROMOS clustering algorithm [60] with a cutoff of 2 Å.

The difference of representative cluster was given as the root mean square deviation (RMSD). The RMSD between two atoms *i* and *j* is defined as:

(3) $$\;{\mathrm{RMSD}}_{i-j}=\sqrt{{\left(\delta_{i-j}\right)}^{2}}\;$$

where δ is the distance between atom *i* and *j* and was computed using vmd [58]. For the comparison of the representative conformations of different clusters from the water and protein simulation the RMSD was computed on all carbon atoms of the dimannosides after alignment of the two conformations.

### 3.6. Free Energy Calculations

The binding free energies ΔG_binding_ of Man, HM, Manα1,2Man, and Manα1,3Man were computed based on the MD trajectories using a selection of residues representing the binding site (Ile13, Asn46, Tyr48, Glu50, Asp54, Arg98, Gln133, Gln135, Tyr137, Asn138, and Asp140). A previous HM FimH simulation [30] was used for the energy calculations. The binding free energy was defined as follows:

(4) $$\Delta G_{\mathrm{binding}}=\;\Delta E_{\mathrm{int}}+\;\Delta G_{\mathrm{solv}}-T\Delta S.$$

The free energy of binding ΔG_binding_ was computed using a hydrid Molecular Mechanics Poisson-Boltzmann Surface Area (MM-PBSA) approach as implemented in g_mmpbsa [61]:

(5) $$\;\Delta G_{\mathrm{binding}}^{*}=\;\Delta E_{\mathrm{int}}+\;\Delta G_{\mathrm{solv}}\;$$

where ΔE_int_ and ΔG_solv_ is the difference in vacuum potential energy and in solvation free energy of the FimH-ligand complex and the FimH and ligand alone in solution, respectively. The vacuum potential energy E_int_ used here is given below:

(6) $$\;E_{\mathrm{int}}={\;E}_{\mathrm{ele}}+{\;E}_{\mathrm{vdw}}\;$$

We only included the energy of nonbonded interactions (electrostatic E_ele_ and van-der-Waals ΔE_vdw_ energetic contributions). Both terms were calculated using the single trajectory approach and the CHARMM36 force field.

The free energy of solvation G_solv_ is defined as the energy required to transfer a solute from vacuum into the solvent. In the here used MM-PBSA approach, it is calculated using an implicit solvent model and a dielectric constant of 4 and 80 was assigned to the protein and water, respectively. G_solv_ has two contributions:

(7) $$\;G_{\mathrm{solv}}={\;G}_{\mathrm{solv}\;\mathrm{NONPOLAR}}+{\;G}_{\mathrm{solv}\;\mathrm{POLAR}}\;$$

The non-polar (non-electrostatic) solvation free energy contribution (ΔG_solv NONPOLAR_) arises from the formation of a cavity within the solvent due to the solution of the solute and from van-der-Waals interactions between the solvent molecules around the cavity and the solute [62]. We used the solvent accessible surface area (SASA) approach, which relates ΔG_solv NONPOLAR_ to the SASA of the solute:

(8) $$\;\Delta G_{solv}=\;\gamma \cdot SASA+\;b\;$$

where γ is a coefficient related to surface tension of the solvent and b is a fitting coefficient. The polar solvation term (ΔG_solv POLAR_) was estimated by solving the Poisson–Boltzmann equation.

## 4. Conclusions

In this study we integrated in a novel way several experimental and theoretical methods to decipher the glycan code for the FimH lectin. In the literature Manα1,2Man has been described early on as shielding the (Manα1,3Man) glycan that is more relevant to strong bacterial (FimH) adhesion and invasion. We could highlight that Manα1,3Man finds a single, well-defined binding position in FimH. Manα1,3Man does not undergo significant conformational changes during the transfer from solvent to the binding site of FimH and neither significantly changes conformation once bound. In contrast, Manα1,2Man stabilizes not as well in the FimH binding site, as evidenced by two alternative conformations populated to 42% and 28% by Manα1,2Man. The difference in binding of the two dimannoses explains the determined preference of FimH towards MGPs exposing Manα1,3Man at the non-reducing end of the high-mannose *N*-glycan.

## Figures and Tables

**Table 1:** Binding affinities and thermodynamic characteristics of the binding of different mono- and dimannoses to FimH. The data used to determine the IC_50_ values and the thermodynamic parameters are shown in Figure 1 and Figure 2, respectively.

Ligand	*Binding affinities*			*Thermodynamic parameters*			
	IC_50_ (ELLSA) [µM]	*K_D_* (ITC) [µM]	*K_D_* (SPR) [µM]		ΔG [kcal mol^−1^]	ΔH [kcal mol^−1^]	TΔS [kcal mol^−1^]
**Manα1,2Man**	55.67 ± 28.8	0.942 ± 0.121	1.260 ^a^		−8.14	−4.26 ± 0.14	3.87
**Manα1,3Man**	22.80 ± 4.75	0.298 ± 0.026	0.320 ^a^		−8.81	−8.23 ± 0.12	0.58
**Man**	52.23 ± 21.71	1.672 ± 0.094 ^b^	2.300 ^c^		−7.80 ^b^	−13.64 ± 0.10 ^a^	−5.84 ^b^
**HM**	0.42 ± 0.05	0.007 ± 0.002 ^b^	0.005 ^c^		−11.00 ^b^	−13.64 ± 0.10 ^a^	−2.65 ^b^

Values are taken from ^a^ [24] ^b^ [32] ^c^ [28].

**Table 2:** Free energies of binding computed by MM-PBSA for different studied mannosides extracted from MD simulations. For more detail, see Section 3.6.

Energy Contributions	Manα1,2Man [kcal/mol]	Manα1,3Man [kcal/mol]	Man [kcal/mol]	HM [kcal/mol]
ΔE_ele_	−156.6 ± 0.6	−187.6 ± 1.1	−153.2 ± 0.5	−157.3 ± 0.7
ΔE_vdw_	−34.5 ± 0.3	−31.9 ± 0.3	−17.1 ± 0.3	−35.0 ± 0.3
ΔE_int_	−191.1± 0.5	−219.5 ± 1.1	−170.3 ± 0.4	−192.3 ± 0.6
ΔG_solv POLAR_	98.3 ± 0.3	123.1 ± 0.8	79.8 ± 0.2	91.3 ± 0.3
ΔG_solv UNPOLAR_	−11.8 ± <0.1	−11.4 ± <0.1	−8.3 ± <0.1	−11.6 ± <0.1
ΔG_solv_	86.5 ± 0.3	111.7 ± 0.8	71.5 ± 0.2	79.7 ± 0.3
ΔG_binding_	−104.6 ± 0.4	−107.7 ± 0.6	−98.8 ± 0.3	−112.6 ± 0.5
